# A long non-coding RNA as a direct vitamin D target transcribed from the antisense strand of the human HSD17B2 locus

**DOI:** 10.1042/BSR20220321

**Published:** 2022-05-27

**Authors:** Yoshiaki Kanemoto, Koichi Nishimura, Akira Hayakawa, Takahiro Sawada, Rei Amano, Jinichi Mori, Tomohiro Kurokawa, Yoshinori Murakami, Shigeaki Kato

**Affiliations:** 1 Department of Medical Epigenetics, Research Institute of Innovative Medicine, Tokiwa Foundation, Iwaki, Fukushima, Japan; 2 Department of Medical Genome Research, Graduate School of Life Science and Engineering, Iryo Sosei University, Iino, Chuo-dai, Iwaki, Fukushima 9708551, Japan; 3 Division of Molecular Pathology, The Institute of Medical Science, The University of Tokyo, Tokyo, Japan; 4 Department of Basic Pathology, School of Medicine, Fukushima Medical University, Fukushima, Fukushima 960-1295, Japan; 5 Department of Hematology, Jyoban Hospital, Tokiwa Foundation, Iwaki, Fukushima, Japan

**Keywords:** eukaryotic gene expression, lncRNA, non-coding RNA, VDR, vitamin D

## Abstract

Vitamin D (VD) exerts a wide variety of actions via gene regulation mediated by the nuclear vitamin D receptor (VDR) under physiological and pathological settings. However, the known target genes of VDR appear unlikely to account for all VD actions. We used *in silico* and transcriptomic approaches in human cell lines to search for non-coding RNAs transcriptionally regulated by VD directly. Four long non-coding RNAs (lncRNAs), but no microRNAs (miRNAs), were found, supported by the presence of consensus VDR-binding motifs in the coding regions. One of these lncRNAs (*AS-HSD17*β*2*) is transcribed from the antisense strand of the *HSD17β2* locus, which is also a direct VD target. *AS-HSD17β2* attenuated *HSD17β2* expression. Thus, *AS-HSD17β2* represents a direct lncRNA target of VD.

## Introduction

Vitamin D (VD) exhibits a wide variety of biological activities under physiological and pathological settings [1,2]. The most well-known function of VD is its role as a hormone-promoting calcium absorption in multiple tissues of the body [3–5]. VD also regulates the cell fate of certain blood cells and cancer cells [6,7]. Most of these VD functions are mediated by a genomic signaling pathway activated by the nuclear vitamin D receptor (VDR), which belongs to a large superfamily (48 members) of steroid/thyroid hormone nuclear receptors. VDR is a DNA-binding and ligand-dependent transcription factor that binds to VD response elements (VDREs) within the promoters of VD target genes [1,2], and also associates with chromatin without stable DNA binding [8,9]. Upon binding to VDREs and related sequences and/or associations with chromatin, activated VDR by ligand binding engages in rapid induction of direct VD target genes [1,2] and late induction of indirect VD target genes. However, the known VD target gene products are unlikely to account for all VD bioactivities [4,6,7,10].

The VD target genes were long believed to comprise only protein-coding genes, transcribed by RNA polymerase II [1,2,7,8]. The direct and canonical VD target genes are defined as those with VDRE-related sequences in their promoters. However, recent human whole-genome sequencing analyses have revealed that numerous non-coding RNAs are transcribed by RNA polymerase II from more than 80% of the genome in a cell-type-specific manner [11,12]. Such findings raise the possibility that some non-coding RNA genes are target genes of DNA-binding transcription factors such as nuclear receptors, which induce transcription via RNA polymerase II, but not RNA polymerase I or III. However, the *in vivo* functions of human non-coding RNAs are difficult to be studied due to species differences; non-coding RNA sequences are generally not conserved among species [12]. Despite this, a few classes of non-coding RNAs have been defined based on function. Small non-coding RNAs such as microRNA (miRNAs) and piwi-interacting RNAs (piRNA) have been characterized as post-transcriptional modulators of mRNA (miRNAs) or regulators of genomic stability (piRNAs) [13]. In contrast with small non-coding RNAs, less is known about long non-coding RNAs (lncRNAs) in terms of their biological functions within the whole genome of different species, and only limited numbers of lncRNAs have been evaluated [11,14,15].

Although miRNAs have been reported to regulate gene expression via VD by modulating mRNA stability and translation levels [16,17], it is unclear if small RNAs and their precursors are transcriptionally (directly) regulated by VD-bound VDR. Neither the presence of VDRE-like motifs nor direct binding of VDR to the promoters of small non-coding RNA gene loci has been reported in higher mammals. Given the wide variety of biological and pathological actions of VD in multiple organs and cellular conditions, we hypothesize that there are non-coding RNAs other than small RNAs that are transcriptionally (i.e., directly) regulated by VD-bound VDR. To test this hypothesis, we performed a genome-wide screening of VD-regulated non-coding RNAs using a human keratinocyte line (HaCaT) with knock-out (KO) of the VDR gene [18,19].

## Materials and methods

### Cell culture and *in vitro* experiments

HaCaT human keratinocytes were cultured in DMEM (high glucose) (Wako, Saitama, Japan) supplemented with 10% fetal bovine serum (Biological Industries, Beit HaEmek, Israel) and penicillin/streptomycin (FUJIFILM Wako Chemicals, Saitama, Japan [19]. VDR-KO HaCaT cells were a kind gift from Dr. S. Sawatsubashi (Tokushima, Japan) [18]. Human prostate cancer cell lines were cultured in RPMI1640 medium (PC3, LNCaP, and CWR22 cells) or DMEM (DU-145 cells) [20,21]. All cells were cultured at 37°C under 5% CO_2_. For quantitative reverse-transcription PCR (qRT-PCR), 1.0 × 10^6^ cells were seeded in a six-well plate and then stimulated with each reagent for the indicated time until harvesting for RNA extraction for qRT-PCR.

### qRT-PCR

Total RNA was isolated from cells using TRIzol™ Reagent (Thermo Fisher Scientific, Carlsbad, CA, U.S.A.) according to the manufacturer’s instructions. cDNA synthesis, PCR, and calculation of the relative RNA expression were performed as previously reported [19–22].

### ChIP assay

The ChIP assays were performed as previously performed [20]. The ChIP-qPCR assays were independently performed more than three-times and similar results were obtained. The data were calculated from the data of the two or three assays, and the representative bands of the PCR products were shown with the molecular markers.

### Luciferase assay

When cells reached 40–50% confluence in 12-well plates, the cells were transfected with the indicated plasmids using the Lipofectamine® 2000 (Thermo Fisher Scientific, Carlsbad, CA, U.S.A.). The total amount of DNA for transfection was adjusted by supplementing with empty vectors. Luciferase activity was determined as previously reported [20,22]. All values are reported as means ± SE from at least three independent experiments.

### Western blotting

The cultured cells were lysed using RIPA buffer (150 mM NaCl, 50 mM Tris-HCl at pH 7.5, 1% NP-40, 0.5% DOC, 0.1% SDS) supplemented with protease inhibitor cocktail tablets (Roche, Basel, Switzerland). We performed SDS-PAGE of 20 μg total proteins from cell lysates. The following antibodies were used for Western blotting [23]: anti-VDR (rabbit, 1:1000; Cell Signaling Technology, Danvers, MA, U.S.A., #12550), anti-HSD17B2 (rabbit, 1:1000; Proteintech, Chicago, IL, U.S.A., 10978-1-AP), and anti-α-tubulin (mouse, 1:1000; Proteintech, Chicago, IL, U.S.A., 66031-1-Ig).

### RNA-seq

RNA-seq library preparation, sequencing, mapping, gene expression analysis, and gene ontology (GO) enrichment analysis were performed by DNAFORM (Yokohama, Kanagawa, Japan). The total RNA quality was assessed by Bioanalyzer (Agilent Technologies, Santa Clara, CA, U.S.A.) to ensure an RNA integrity number greater than 8.0. After poly (A) + RNA enrichment using the NEB Next Poly(A) mRNA Magnetic Isolation Module (New England BioLabs, Ipswich, MA, U.S.A.), double-stranded cDNA libraries (RNA-seq libraries) were prepared using SMARTer® Stranded RNA-Seq Kit v2 (Takara Bio, Kusatsu, Shiga, Japan), according to the manufacturer’s instruction. RNA-seq libraries were sequenced using paired end reads (50 and 25 nucleotides: reads 1 and 2, respectively) on the NextSeq 500 instrument (Illumina, San Diego, CA, U.S.A.). The obtained raw reads were trimmed and quality-filtered using the Trim Galore! (version 0.4.4), Trimmomatic (version 0.36), and cutadapt (version 1.16) software. Trimmed reads were then mapped to the human GRCh37.p13 genome using STAR (version 2.7.2b) [24]. Reads of annotated genes were counted using featureCounts (version 1.6.1). FPKM values were calculated from mapped reads by normalizing to total counts and transcript length. Differentially expressed genes were detected using the DESeq2 package (version 1.20.0). The list of differentially expressed genes detected by DESeq2 (base mean > 5 and fold-change < 0.25, or base mean > 5 and fold-change > 4) (accession#: GSE178702) was subjected to GO enrichment analysis using the cluster Profiler package [25].

### NET-CAGE

CAGE library preparation, sequencing, mapping, and motif expression and discovery analysis were performed by DNAFORM (Yokohama, Kanagawa, Japan). Total RNA quality was assessed by Bioanalyzer (Agilent Technologies, Santa Clara, CA, U.S.A.) to ensure an RNA integrity number greater than 7.0. The cDNAs were synthesized from total RNA using random primers. The ribose diols in the 5’ cap structures of RNAs were oxidized, and then biotinylated. The biotinylated RNA/cDNAs were selected by streptavidin beads (cap-trapping). After RNA digestion by RNaseONE/H and adaptor ligation to both ends of cDNA, double-stranded cDNA libraries (CAGE libraries) were constructed. CAGE libraries were sequenced using single end reads of 75nt on a NextSeq 500 instrument (Illumina, San Diego, CA, U.S.A.). Obtained reads (CAGE tags) were mapped to the human GRCh37.hg19 genome using BWA (version 0.5.9). Unmapped reads were then mapped by HISAT2 (version 2.0.5). CAGE tag clustering, detection of differential expressed genes, and motif discovery were performed by pipeline RECLU [26]. Tag count data were clustered using the modified Paraclu program. Clusters with count per million (CPM) < 0.1 were discarded. Regions that have 90% overlap between replicates were extracted by BEDtools (version 2.12.0). The cluster with irreproducible discovery rate (IDR) ≥ 0.1 and clusters longer than 200 bp were discarded. Differentially expressed genes were detected using the edgeR package (version 3.22.5). For motif analysis, the genomic DNA sequence of the region from 200 bp upstream to 50 bp downstream of differentially expressed CAGE peaks were subjected to *de novo* motif discovery tools: AMD, GLAM2, DREME, and Weeder. The occurrences of the motifs were examined by the FIMO. The similarity of consensus motifs and the motifs in database JASPAR CORE 2016 vertebrates were evaluated by Tomtom. The list of differentially expressed genes detected by RECLU with false discovery rate (FDR) ≤0.05 were used for GO enrichment analysis by clusterProfiler package [25] and registered as GSE179017.

### ATAC-seq

ATAC sequence library preparation, sequencing, mapping, gene expression, and GO enrichment analysis were performed by DNAFORM (Yokohama, Kanagawa, Japan). Fragmentation and amplification of the ATAC-seq libraries were conducted according to Buenrostro et al. (2015) [25]. Briefly, approximately 100000 cells were lysed and subjected to the transposition reaction using Tn5 Transposase (Illumina, FC121-1030) at 37°C for 30 min. The reaction liquid was purified using the Qiagen MinElute PCR Purification Kit. Then, five cycles of PCR were conducted using NEBNext Q5 Hot Start HiFi PCR Master Mix (New England Biolabs) and custom Nextera PCR primers [27,28]. Additional PCR cycles were performed up to the results of qPCR of the partly amplified products [27]. The PCR products were purified using Agencourt AMPure XP beads (Beckman Coulter, Brea, CA, U.S.A.: A63881) via double-size selection (left ratio: 1.4, right ratio: 0.5), according to the manufacturer’s protocol. Paired-end sequencing was performed on the Illumina HiSeq sequencer. Reads were mapped to the hg38 reference sequence using the Burrows–Wheeler aligner (ver. 0.7.17-r1188), and duplicate reads were removed using Picard (ver. 2.18.16). Peak calling was performed using PePr (ver. 1.1.24) with the default parameters. Peak annotations were obtained by HOMER (ver. 4.9.1) using the default settings. Known motifs and *de novo* consensus motifs within the central 200 bp of the obtained peaks were searched by the HOMER using the default settings and registered as GSE191099.

### Statistical analysis and reproducibility

Comparison of mean values was conducted using Mann--Whitney U test. Significant differences between experimental groups are indicated with asterisks as follows: *P<0.05 and **P<0.01. All values are reported as means ± SD from at least three independent experiments [22].

## Results

### Expression of VD-regulated non-coding RNA genes in human keratinocytes

**Figure 1:**
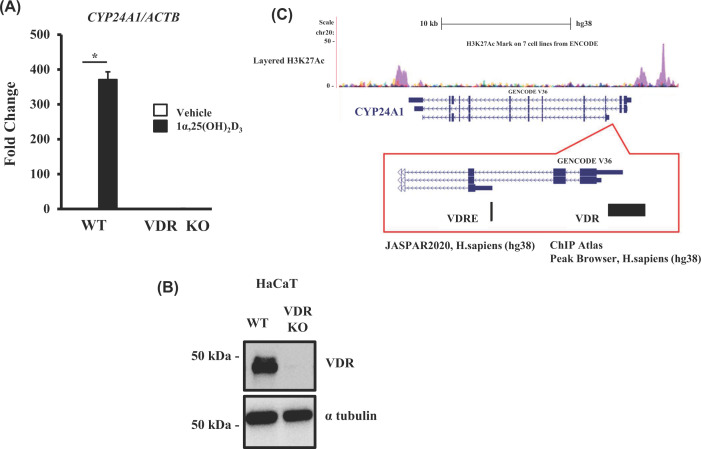
Human CYP24A1 as a direct vitamin D target gene in HaCaT cells (**A**) Robust induction of *CYP24A1* by Vitamin D-mediated VDR. The wild-type (WT) HaCaT cells and *VDR*-KO HaCaT cells (VDR-KO) were treated with 1α,25(OH)_2_D_3_ for 8 h, and the extracted RNA was subjected to qRT-PCR. Data are expressed as the mean ± SE of three samples. (**B**) No VDR protein in the VDR-KO cells. Western blotting of VDR in the WT and *VDR*-KO cells. (**C**) The locus of human *CYP24A1*. Both VDRE-related motifs by JASPAR2020 and VDR-binding peaks registered in ChIP-atlas are shown.

**Figure 2:**
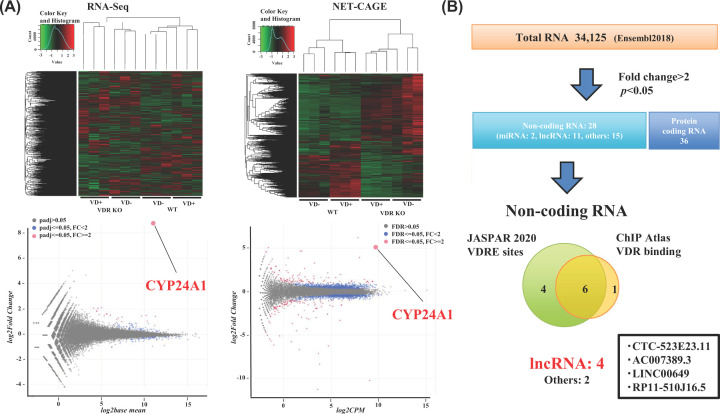
Transcriptome analysis of vitamin D-regulated non-coding RNAs in human keratinocytes (HaCaT cells) (**A**) Transcriptome analysis of vitamin D-regulated genes.*VDR*-KO and WT HaCaT cells treated with 1α,25(OH)_2_D_3_ for 8 h were subjected to RNA-seq (GSE178702) and NET-CAGE (GSE179017), and a heatmap (upper panel) and MA-plot are shown in the lower panel. *CYP24A1* is shown as the reference indicator for up-regulated genes by vitamin D. (**B**) *In silico* mining of VD target non-coding RNAs. A total of 28 non-coding RNAs and 36 mRNAs with greater than twofold up-regulated expression were selected from the differential gene expression analysis. *In silico* screening of VDRE-related motifs, according to the JASPAR 2020 database, and of VDR-binding peaks, according to the ChIP-Atlas, in non-coding RNA loci of the human genome was performed. The six non-coding RNA candidates, but not the two miRNAs, encompass the registered VDRE-related motifs and VDR-binding peaks. The four non-coding RNAs are registered lncRNAs (CTC-523E23.11, AC007389.3, LINC00649, and RP11-510J16.5).

To determine whether human non-coding RNAs are transcriptionally regulated by VD, we used the HaCaT keratinocyte line, as skin is a major organ involved in VD biosynthesis. We previously showed in VDR-KO HaCaT cells that VD transcriptionally regulates the known VD target gene *CYP24A1* [18,19]. Therefore, using VDR-KO HaCaT cells to confirm the candidate genes as direct VDR target genes, we performed transcriptome analysis by RNA-seq as well as NET-CAGE to detect nascent transcripts from both DNA strands. The VDR-KO and wild-type cells (Figure 1A) were treated with 1α,25(OH)_2_D_3_ for 8 h, and the extracted RNA was subjected to transcriptome analysis. The VDR dependency of the VD response was evaluated using CYP24A1 expression as a positive indicator. As reported previously [19], robust induction of CYP24A1 by VD was observed in the wild-type cells but not in the VDR-KO cells (Figure 1B), verifying gene regulation by VD-bound VDR. Both RNA-seq and NET-CAGE showed that numerous transcripts were up-regulated and down-regulated by VD, as illustrated in the heatmap and MA-plot in Figure 2A. *In silico*, we extracted VD-regulated non-coding RNA gene candidates from human genome databases (Ensembl 2018 and FANTOM CAGE project). Among 34125 transcripts expressed in HaCaT cells, the numbers of protein-coding and non-coding mRNAs were 739 and 91, respectively (Figure 2B).

### *In silico* data mining of candidate VD non-coding RNA targets

We characterized the 91 non-coding RNAs obtained by *in silico* data mining [21,22] as direct VD targets if they were found to be transcriptionally regulated by VD-bound VDR. Of these, 28 non-coding RNAs, including two miRNAs and 11 lncRNAs, with greater than twofold up-regulated expression, were selected. To evaluate the expression of these 28 non-coding RNAs in response to VD, we performed NET-CAGE, which can detect the nascent RNAs transcribing the non-coding RNAs from both strands. After close assessment of the 28 gene loci, all appeared to produce nascent RNAs from either one strand or both strands in response to VD, consistent with the RNA-seq data (Figure 2A).

**Figure 3:**
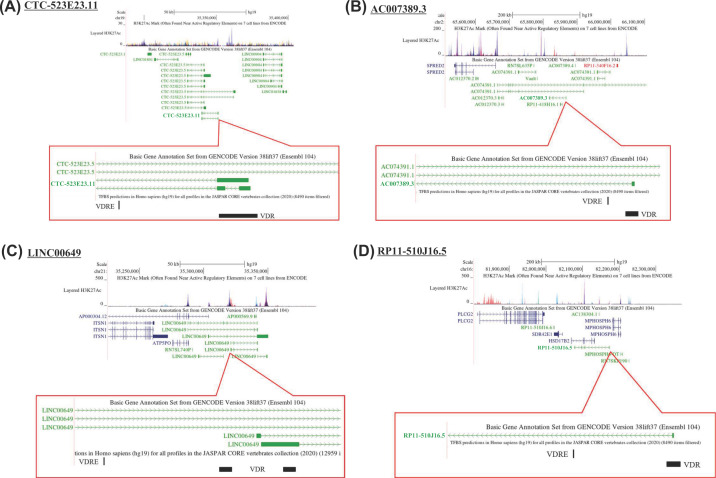
The gene loci of the four lncRNAs regulated by vitamin D (**A**) The gene locus for CTC-523E23.11, (**B**) for AC007389.3, (**C**) for LINC00649, and (**D**) for RP11-510J16.5. The registered peaks for H3K27Ac are shown in the upper panels, and VDR-binding regions (the ChIP-Atlas) and VDRE-related motifs (the JASPAR 2020) are displayed in the lower panels.

Next, the loci of the non-coding RNAs were assessed *in silico* for the registered VDR-binding peaks, according to the ChIP-Atlas, and for VDRE-related motifs, according to the JASPAR 2020 database. Neither VDR-binding peaks nor VDRE-related motifs were detected in the gene loci of the two miRNAs or in their adjacent ∼10 kb downstream/upstream regions. In contrast, the gene loci of the four candidate lncRNAs were found to harbor both for the registered VDR-binding peaks and VDRE-related motifs (Figure 3A–D), similar to the gene locus of CYP24A1 (Figure 1C).

### A lncRNA (AS-HSD17β2) as a direct VD target

**Figure 4:**
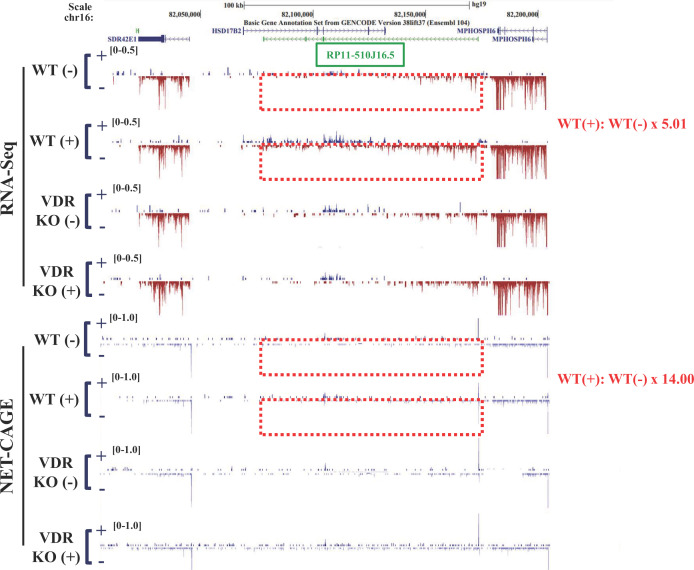
A lncRNA (*RP11-510J16.5/AS-HSD17β2*) is transcribed from the antisense strand of the HSD17β2 locus The genomic structure of human *HSD17β2* on chromosome 16 is shown in the upper panel. The peaks representing transcripts detected by RNA-seq and NET-CAGE in wild-type HaCaT cells treated without [WT (-)] or with [WT (+)] and *VDR*-KO cells 1α,25(OH)_2_D_3_ for 8 h are shown in the lower panel. Compared with no 1α,25(OH)_2_D_3_ treatment, 1α,25(OH)_2_D_3_ induced an approximately fivefold (from RNA-seq data) and 14-fold (from NET-CAGE data) up-regulation of the average transcript signal of *HSD17β2* and *AS-HSD17β2* (*RP11-510J16.5*), calculated from the MA-plots in Figure 2A.

**Figure 5:**
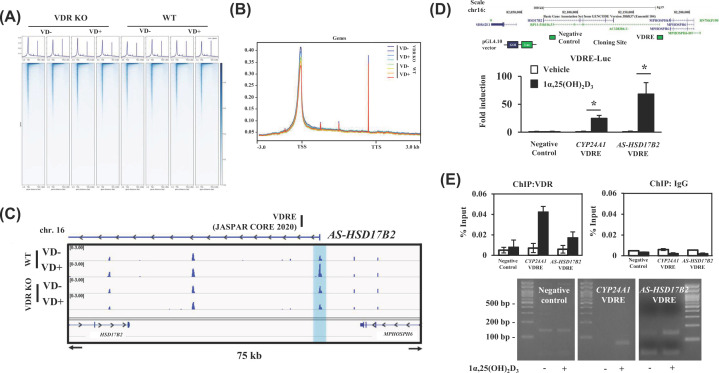
Vitamin D-induced and VDR-dependent chromatin reconfiguration at the human *HSD17β2/AS-HSD17β2* gene locus (**A**) Heatmap and mapping profile of ATAC-seq data. The analysis of ATAC-seq data by deepTools (GSE191099) is shown. All genes with one or more reads were mapped by averaging the read depth at each position over a range of 3 kb upstream of the transcription start site (TSS) to 3 kb downstream of the transcription termination site (TTS) and normalizing to the ATAC signal. Genes with one or more reads are indicated by a single horizontal line, and read depths from 3 kb upstream of the TSS to 3 kb downstream of TTS are indicated. (**B**) Comparison of each mapping profile among the samples. (**C**) IGV tracks of ATAC-seq data from a representative locus, AS-HSD17B2 (scale 0–3.0), are presented. The blue box represents the TSS of *AS-HSD17B2*. (**D**) Identification of a VDRE at the human *HSD17β2/AS-HSD17β2* gene locus. An *HSD17β2/AS-HSD17β2* VDRE at the human *HSD17β2/AS-HSD17β2* gene locus (upper panel) was assessed by a luciferase reporter assay in HaCaT cells treated with or without 1α,25(OH)_2_D_3_ for 24 h (lower panel). A region in the gene locus was used as a negative control as depicted in the panel. The assay was independently performed three-times. Data are expressed as the mean ± SE of three samples. (**E**) Vitamin D-induced association of VDR with *HSD17β2/AS-HSD17β2* VDRE. The ChIP-qPCR assay was performed in HaCaT cells. The known VDRE in the *CYP24A1* gene promoter was used as a positive control. The ChIP-qPCR assay (37 PCR cycles for negative control and *CYP24A1* VDRE, 35 cycles for *AS-HSD17β2* VDRE) was independently performed more than three-times and similar results were obtained. Data are expressed as the mean ± SD of the two or three samples, and the representative bands of the PCR products were shown together with the molecular markers in the panels.

The coding region of one of the four lncRNAs mapped to the gene locus encoding the HSD17β2 enzyme, which catalyzes sex steroid hormones [29]. This lncRNA (*RP11-510J16.5*) is hereafter referred to as *AS-HSD17β2*. NET-CAGE confirmed the expression of *HSD17β2* mRNA and *AS-HSD17β2* lncRNA from each strand at the same gene locus and in response to VD (Figure 4). VDR dependency in the VD response was confirmed by means of the VDR-KO cells (Figure 4). The VDR-binding peaks are located within 1–5 kb from the VDRE-related motifs in the adjacent chromatin regions, but they did not overlap with those motifs (Figure 3D). As the chromatin sites associated with given DNA-binding transcription factors often do not overlap with the consensus-binding motifs in the gene promoters, conceivably due to chromatin looping, we presumed that the regulatory region adjacent to the *AS-HSD17β2* locus forms a specific tertiary structure facilitating VD-regulated transcription. ATAC-seq was performed to assess the effect of alterations in chromatin configuration on this gene locus in HaCaT cells treated with or without 1α,25(OH)_2_D_3_ for 8 h. Although the whole genome structure did not appear to be significantly regulated by VDR-mediated VD signaling (Figure 5A,B), this gene locus was found to be locally regulated by VD at the chromatin structure level, and the VDR dependency was confirmed in VDR-KO cells (Figure 5C). Thus, we presume that at this gene locus, chromatin structure is reorganized by VD to enable efficient transcription.

We then evaluated whether this VDRE-related motif (*AS-HSD17β2* VDRE) indeed behaves as a VDRE. In a luciferase reporter assay (Figure 5D), robust VD-responsive enhancer activity was detected at the *AS-HSD17β2* VDRE compared with a known VDRE in *CYP24A1* in human keratinocytes [30]. Moreover, in HaCaT cells, efficient binding of endogenous VDR to the *AS-HSD17β2* VDRE was observed by the ChIP-qPCR assay (Figure 5E), though the *AS-HSD17β2* VDRE is not overlapped with the registered peaks of VDR binding in the ChIP-atlas. Thus, together with the RNA-seq and NET-CAGE findings, we propose that *AS-HSD17β2* is a direct lncRNA target of VD.

**Figure 6:**
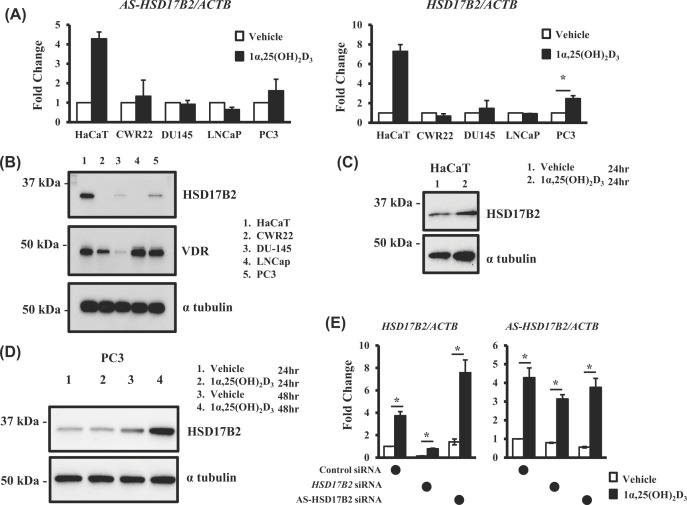
Vitamin D-induced expressions of *HSD17β2* and *AS-HSD17β2* and the attenuating function of *AS-HSD17β2* for *HSD17β2* expression (**A-D**) Vitamin D-induced expressions of *HSD17β2* and *AS-HSD17β2*. Expression of *HSD17β2* and *AS-HSD17β2* were assessed in HaCaT cells and in prostate cancer cells at protein and transcript levels estimated by Western blotting and qRT-PCR, respectively. (**E**) Attenuation of *HSD17β2* gene expression by *AS-HSD17β2*. A knockdown assay using siRNAs specific to each strand of the human *HSD17β2*/*AS-HSD17β2* locus was performed in HaCaT cells treated with or without 1α,25(OH)_2_D_3_ for 8 h. The assay was independently performed four-times. The cells were subjected to qRT-PCR. Data are expressed as the mean ± SD of three samples.

Since the *AS-HSD17β2* mRNA level was regulated by VD, and the AS-HSD17β2 VDRE is a potential VDRE for the *HSD17β2* gene as well, we evaluated whether *HSD17β2* is a direct VD target. In HaCaT cells, the response of *HSD17β2* transcription to VD was evident by the qRT-PCR assay, as expected (Figure 6A). *HSD17β2* expression showed a similar VD response (Figure 6A); however, qRT-PCR cannot distinguish the two different strands of a gene locus. Therefore, we assessed the VD response of HSD17β2 at the protein level by Western blotting (Figure 6B). An increase in HSD17β2 protein expression after VD treatment was detected (Figure 6C), suggesting that HSD17β2 is a direct VD target gene.

As a role of HSD17β2 in prostate cancer development has been reported [31], we next evaluated whether HSD17β2 and AS- HSD17β2 are transcriptionally regulated by VD in prostate cancer. Among the tested prostate cancer cell lines (CWR22, DU-145, LNCaP, and PC3), the HSD17β2 protein level in PC3 cells was relevant to that in HaCaT cells (Figure 6B). When the PC3 cells were treated with 1α,25(OH)_2_D_3_ increased protein and transcript levels of HS17β2 were observed (Figure 6D). Thus, these findings suggest that HS17β2 expression is affected by VD, at least in certain prostate cancer cell types. Likewise, up-regulation of *AS-HSD17β2* by VD was seen in the CWR22 and PC3 cells (Figure 6C), confirming our idea that *AS-HSD17β*2 is a VD direct target in the prostate cancer cells other than keratinocyte cells.

### AS-HSD17β2 attenuation of HSD17β2 expression

As *AS-HSD17β2* transcripts and HSD17β2 mRNA are transcribed from the same gene locus, we assessed the mutual relationship between their expression levels in HaCaT cells. A knockdown experiment was performed using siRNAs selectively targeting each transcript derived from exon 4 of *HSD17β2*. The *AS-HSD17β2* expression level was slightly reduced by the siRNA targeting *AS-HSD17β2* transcript but was not affected by the siRNA-targeting HSD17β2 mRNA. However, knockdown of *AS-HSD17β2* transcript effectively up-regulated HSD17β2 mRNA expression (Figure 6E), and VD responsiveness was retained. Thus, these findings suggest that *AS-HSD17β2* transcript attenuates the expression of HSD17β2 mRNA.

## Discussion

In the present study, we found numerous non-coding RNA gene candidates regulated by VD, some via VDR, by using RNA sequencing (RNA-seq) and native elongating transcript–cap analysis of gene expression (NET-CAGE), together with an *in silico* search of human miRNAs and lncRNAs and of VDR-binding sites in public databases. In HaCaT cells, the 91 non-coding RNA candidates were up-regulated by VD, while non-coding RNAs down-regulated by VD remain to be identified (Figure 2A,B). Two miRNAs were present among these non-coding RNAs (Figure 2B), and miRNAs are known to modulate many biological events by inhibiting the translation of, and destabilizing, the target mRNAs [13,32]. However, they are unlikely to be direct VD targets, since neither VDR-binding sites from the database nor VDRE-like motifs were found in their gene loci or 10-kb adjacent regions. Of these non-coding RNA targets, VDR-binding sites and VDRE-related sequences registered in the databases were absent in the gene loci of the miRNAs. In the contrast, the gene loci of four lncRNAs matched this criterion. Two classes of non-coding RNAs (lncRNAs and enhancer RNAs) have already been illustrated to exert biological functions [14,33]. Enhancer RNAs serve as transcriptional and epigenetic regulators by dynamic reconfiguration of the chromatin environment [34,35]. Consistent with our previous reports [21,22], we recently detected candidate VD-induced eRNAs in the VD-responsive gene promoter of CYP24A in HaCaT cells [19]. The present results suggest that certain lncRNAs other than eRNAs are regulated by VD [33,34]. In our tested cell lines derived from human keratinocytes, four lncRNAs were transcriptionally induced by VD-bound VDR (Figures 2 and 4). The VDRE-like motif located in the AS-HSD17β2 gene locus acted as a VDRE according to the luciferase reporter assay (Figure 5D), and its VDRE activity was higher than that of the known VDRE in the CYP24A1 gene promoter [29,36]. Furthermore, binding of endogenous VDR to this *AS-HSD17β2* VDRE was detected by the ChIP-qPCR assay in HaCaT cells (Figure 5E). Consistently, the regulation of the *AS-HSD17β2* gene by VD was also seen in the other cell lines derived from prostate cancer (CWR22 and PC3 cells) (Figure 6). Additional direct VD lncRNA targets may be identified in other cell types and tissues using our same approach. Considering the significance of VD signaling in pathological settings [4,10,37,38], VD-regulated lncRNAs might play critical roles in cancer onset and development. However, genetic manipulation to assess the *in vivo* function of the four lncRNAs identified here is difficult in animal models, since the genomic sequences encoding these lncRNAs in humans are not conserved in rodents, similar to most non-coding RNAs [11,12,14]. Ectopic expression of a given human lncRNA in animal models may provide clues to address this issue.

We revealed the biological function of *AS-HSD17β2* in terms of *HSD17β2* gene regulation. When *AS-HSD17β2* expression was knocked down by siRNA in HaCaT cells, *HSD17β2* expression was up-regulated in the presence of VD (Figure 6E). In contrast, when HSD17β2 expression was knocked down, such up-regulation was not seen for *AS-HSD17β2* expression. Thus, the *AS-HSD17β2* transcript appears to attenuate HSD17β2 mRNA expression, although the mechanism remains to be determined. Given its longer length compared with HSD17β2 mRNA, *AS-HSD17β2* transcript(s) might serve as a miRNA precursor to degrade HSD17β2 mRNA. This idea is supported by recent characterization of miRNAs transcribed from the antisense strands of introns of the genes encoding a neural tau protein and the ubiquitin E3 ligase Wwp protein [39,40]. As both *AS-HSD17β2* and *HSD17β2* transcription were induced by VD, *AS-HSD17β2* may act as a VD sensor to suppress an excess of VD-induced expression of HSD17β2 mRNA in cells expressing VDR.

In the characterization of *AS-HSD17β2* as a direct VD target lncRNA, *HSD17β2* was found to be a novel VD target gene. The incidence of prostate cancer was suggested to be closely associated with VD nutrition status in a large number of epidemiological studies [41], and VD is considered beneficial for preventing prostate cancer [42]; however, the molecular basis of this VD effect is poorly understood. In this respect, VD-mediated gene regulation of *HSD17β2* and *AS-HSD17β2* is interesting, but its significance in prostate cancer awaits further study. Consistent with the putative enzymatic activity of HSD17β2 in sex steroid hormone biosynthesis, a recent report suggested that HSD17β2 drives prostate cancer development based on clinical observations of HSD17β2 overexpression in prostate tumors [41,42]. In the present study, we also observed HSD17β2 expression at the mRNA and protein levels in the CWR22 and PC3, but not DU-145 and LNCaP, prostate cancer cell lines, and its co-expression with *AS-HSD17β2* transcript(s) (Figure 6). Since the transcription of *AS-HSD17β2* occurs from the antisense strand of the *HSD17β2* locus at multiple transcription start sites (Figures 4 and 5), it is possible that decreased *AS-HSD17β2* expression due to either reduced promoter activity or a different transcription start site leads to overexpression of HSD17β2 in prostate tumors.

Though we could provide an evidence that four lncRNA genes are direct VD targets, only two types of human cell lines (keratinocytes and prostate cancer) were applied for the present study. Similarly, the gene regulation of HSD17β2 by VD has been assessed in prostate cell lines. Thus, the VD action for the genes of four lncRNAs and HSD17β2 in human intact tissues including prostate tumors still remain to be tested.

In summary, our results suggest that lncRNAs are direct non-coding RNA targets of VD and play a role in facilitating VD signaling, and further characterization of the roles of HSD17β2 and *AS-HSD17β2* in prostate cancer development is clearly needed.

## Data Availability

The data of the RNA-seq are registered as accession#: GSE178702, and those of the NET-CAGE and ATAC-seq are the accession# :GSE179017 and GSE191099, respectively. The other datasets are available upon reasonable request from the corresponding author.

## Abbreviations

eRNA: enhancer RNA
HSD17β2: Hydroxysteroid 17-Beta Dehydrogenase 2
KO: knock-out
lncRNA: long non-coding RNA
miRNAs: microRNA
piRNA: piwi-interacting RNAs
qRT-PCR: quantitative reverse-transcription polymerase chain reaction
VD: vitamin D
VDR: vitamin D receptor
VDRE: vitamin D response element
